# The amino acid transporter Slc7a5 regulates the mTOR pathway and is required for granule cell development

**DOI:** 10.1093/hmg/ddaa186

**Published:** 2020-08-21

**Authors:** Aidan M Sokolov, Jennie C Holmberg, David M Feliciano

**Affiliations:** Department of Biological Sciences, Clemson University, Clemson, SC 29634-0314, USA; Department of Biological Sciences, Clemson University, Clemson, SC 29634-0314, USA; Department of Biological Sciences, Clemson University, Clemson, SC 29634-0314, USA

## Abstract

Pathogenic mutations in the solute carrier family 7 member 5 (SLC7A5) gene, which encodes an amino acid transporter cause microcephaly and seizures, yet the mechanisms responsible for these phenotypes are unclear. Models have demonstrated that Slc7a5 deletion is embryonic lethal and that these embryos lack a fully formed telencephalon. This phenotype is similar to that of mammalian target of rapamycin (mTOR) protein kinase deletion or mTOR inhibition. Notably, in many cells, Slc7a5 import of amino acids is required to maintain mTOR activity. Slc7a5 is present within neurogenic regions during embryogenesis, is found in cultured neurons and can modulate neuronal electrophysiological properties. However, Slc7a5 is also highly expressed within endothelial cells of the blood–brain barrier where removal in conditional mice leads to severe behavioral defects and non-cell autonomous changes in neurons. Therefore, the extent that neural Slc7a5 is required for development is unclear. Here, subventricular zone neural stem cells that generate olfactory bulb granule cell neurons were electroporated with SLC7A5 or Slc7a5 short hairpin RNA encoding plasmids. Although early phases of neural development were unaltered, Slc7a5 knockdown effected late phases of GC dendrite maturation and survival. Slc7a5 knockdown also decreased mTOR pathway activity. Ras homolog enriched in brain, an mTOR activator, rescued the effect of Slc7a5 knockdown on mTOR pathway activity and dendrite arbors. The data presented here demonstrate that Slc7a5 is required for GC mTOR pathway activity, maturation and survival, which may help explain why Slc7a5 mutations prevent normal brain development and function.

## Introduction

Inactivating mutations in the solute carrier family 7 member 5 (SLC7A5) gene lead to a neurodevelopmental disorder characterized by microcephaly, seizures and neuropsychiatric manifestations (1). SLC7A5 single nucleotide polymorphisms, copy number variants and altered expression are also associated with a wide range of neuropathologies (2–7).

Slc7a5 deletion is embryonic lethal and leads to robust neural defects in mice (8, 9). Slc7a5 is expressed within embryonic cultures of cortical neurons, the developing neural tube and forebrain, and in postnatal neurogenic regions (9–11). Slc7a5 also regulates Kv1.2 dendritic voltage-gated potassium channels in developing hippocampal neurons (12). Thus, at first appearance, Slc7a5 seems to play an important role in nervous system development. However, expression is notably absent from mature neurons in the adult nervous system where it appears confined to endothelial cells of the blood–brain barrier (BBB) (1, 13). Slc7a5 deletion from the developing BBB causes behavioral defects and changes in neuronal excitatory/inhibitory balance in mice (1). Notably, mice with *Slc7a5* deletion from the BBB had no reported brain architectural defects whereas patients with *Slc7a5* mutations and *Slc7a5* knockout mouse brains are severely altered (1, 9). Therefore, whether Slc7a5 has a cell-autonomous role in neural cells requires further clarification.

Slc7a5 is an evolutionarily conserved transporter that facilitates cellular uptake of neutral amino acids (14–16). Slc7a5 is associated with Slc3a2 in a heteromeric complex and together function as an amino acid antiporter (17). Notably, Slc7a5 transports leucine into cells (14, 17). Leucine is an anabolic amino acid that stimulates the protein kinase, mammalian target of rapamycin (mTOR), protein translation and cell growth (18–26). mTOR is the catalytic component of two complexes, mammalian target of rapamycin complex 1 (mTORC1) and mammalian target of rapamycin complex 2 (mTORC2) (26). mTORC1 controls translation of 5′ Terminal Oligo Pyrimidine containing mRNAs by phosphorylating eukaryotic initiation factor 4E (eIF4E) binding protein, an inhibitor of eIF4E (27–29). mTORC1 also phosphorylates and thereby activates p70S6 kinase, which subsequently phosphorylates the ribosomal S6 subunit (S6) at Ser 240/244 (29–31). Critically, mTORC1 regulates neuron morphology including dendrite complexity (32–36).

Subventricular zone (SVZ) neural stem cells (NSCs) that reside along the lateral ventricles comprise the largest postnatal neurogenic compartment in the brain (37). SVZ NSCs generate neuroblasts that migrate through the rostral migratory stream (RMS) into the olfactory bulb (OB), where ~94% mature into inhibitory granule cells (GCs) (38–42). Here, the role of Slc7a5 in SVZ neurogenesis and GC maturation was examined. RNA interference resulting in Slc7a5 knockdown *in vivo* dramatically altered late phases of GC dendrite maturation and GC survival. Furthermore, Slc7a5 knockdown reduced mTOR pathway activity. Importantly, mTORC1 activation rescued Slc7a5 knockdown effects on GC maturation. The results of this study provide evidence that Slc7a5 regulates GC mTOR pathway activity and is required for late phases of dendrite maturation and survival.

## Results

### *In vitro* and *in vivo* genetic Slc7a5 manipulation

**Figure 1:**
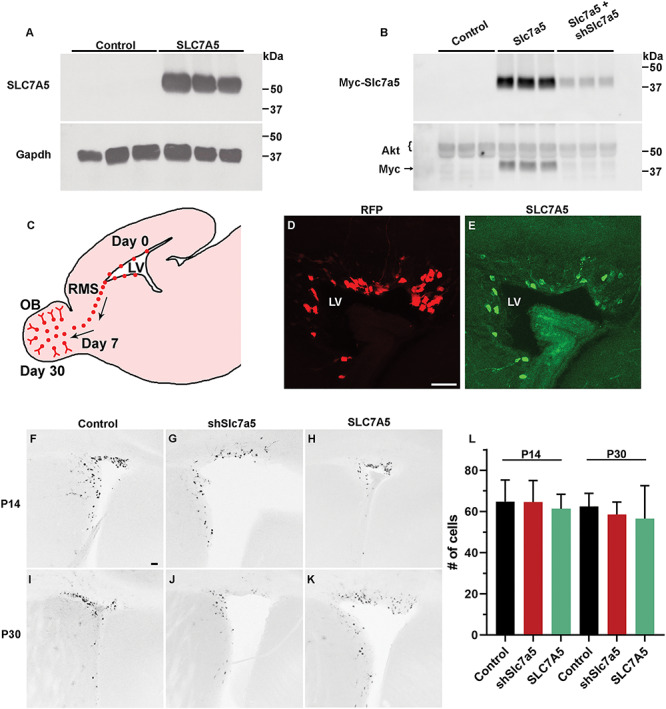
Control of Slc7a5 expression. (**A**) Western blot for SLC7A5 following control or SLC7A5 plasmid transfection of Neuro-2a cells (*N* = 6, 6). (**B**) Western blot for myc from lysates of Neuro-2a cells transfected with control, myc-Slc7a5 and control, or myc-Slc7a5 and Slc7a5 shRNA plasmids (*N* = 3, 3, 3). (**C**) Schematic diagram representing a sagittal section of the mouse brain. The diagram depicts electroporated (red) NSCs surrounding the LVs that generate neuroblasts that migrate through the RMS and enter into the OB 7 days post-electroporation and become mature GCs 30 days post-electroporation. (**D** and **E**) ×20 image of RFP+ cells co-electroporated with SLC7A5 in the SVZ and stained for SLC7A5. Scalebar = 50 μm. (**F**–**K**) ×5 images of P14 and P30 SVZs following P0 co-electroporation with RFP and control, shSlc7a5, or SLC7A5 plasmids. P14 control, *N* = 6; shSlc7a5, *N* = 6; or SLC7A5, *N* = 3. P30 control, *N* = 7; shSlc7a5, *N* = 8; or SLC7A5, *N* = 4 scalebar = 100 μm. (**L**) Quantification of F–K.

Neuro-2a cells are a cancerous cell-line of neural origin that have high transfection efficiency and are routinely used to study the mTOR pathway (43, 44). Neuro-2a cells were transfected with the human form of *SLC7A5* under the control of a CAG promoter. This plasmid increased SLC7A5 expression by ~ 100-fold as determined by western blot using an antibody that detects only the human form of SLC7A5 (*P* < 0.001) (Fig. 1A). Neuro-2a cells were also transfected with a plasmid encoding mouse *Slc7a5* fused to a myc-tag epitope because no commercially available antibodies to Slc7a5 faithfully recognized mouse Slc7a5 (data not shown). Slc7a5 was increased (*P* < 0.0001) as determined by western blot using an antibody to myc (Fig. 1B). In addition, a plasmid encoding short hairpin (sh) RNA to Slc7a5 (shSlc7a5) was co-transfected with mouse *Slc7a5*. Short hairpin RNA (shRNA) mediated knockdown of Slc7a5 was validated by western blot with the antibody to myc and reduced Slc7a5 by ~ 71% (*P* < 0.0001) (Fig. 1B). These results mirrored the effect of shSlc7a5 on myc-Slc7a5 when compared with cells transfected with a control shRNA and *Slc7a5* (Fig. S1A). Moreover, shSlc7a5 reduced leucine import (Fig. S1B).

Postnatal (P) day 0 mouse SVZ NSCs were electroporated with red fluorescent protein (RFP) and empty vector control, shSlc7a5 or SLC7A5 encoding plasmids. SVZ NSCs give rise to neuroblasts that migrate along the RMS, enter into the OB ~ 7 days later, take an additional 7 days to extend dendrites into the GC layer, and take ~ 16 more days to fully mature (Fig. 1C) (45). Brains were therefore harvested and examined at P14 and P30. Immunohistochemistry with an antibody to human SLC7A5 revealed robust expression in RFP and SLC7A5 co-electroporated cells in the SVZ (Fig. 1D and E). The number of SLC7A5 or shSlc7a5 cells in the SVZ did not change in comparison to controls at P14 or P30 (Fig. 1F–L). Slc7a5 knockdown was confirmed to persist *in vivo* at P30 (Fig. S2A) (9). Slc7a5 protein is expressed within the SVZ, RMS and OB (11). *In situ* hybridization (ISH) from the Allen Institute confirmed Slc7a5 expression in OBs (Fig. S2B). Slc7a5 expression was confirmed by reverse transcription-polymerase chain reaction (RT-PCR) (Fig. S2C). bDNA fluorescence *in situ* hybridization (FISH) demonstrated that Slc7a5 mRNA was present in the GC layer (Fig. S2D and G). bDNA FISH was then performed in RFP electroporated brains which confirmed expression within GCs (Fig. S2E, F, H and I). OBs of the electroporated mice were next examined because neurons do not develop properly in Slc7a5 knockout mice and because patients with Slc7a5 mutations have microcephaly (1, 9).

### Slc7a5 is required for late phases of dendrite maturation

**Figure 2:**
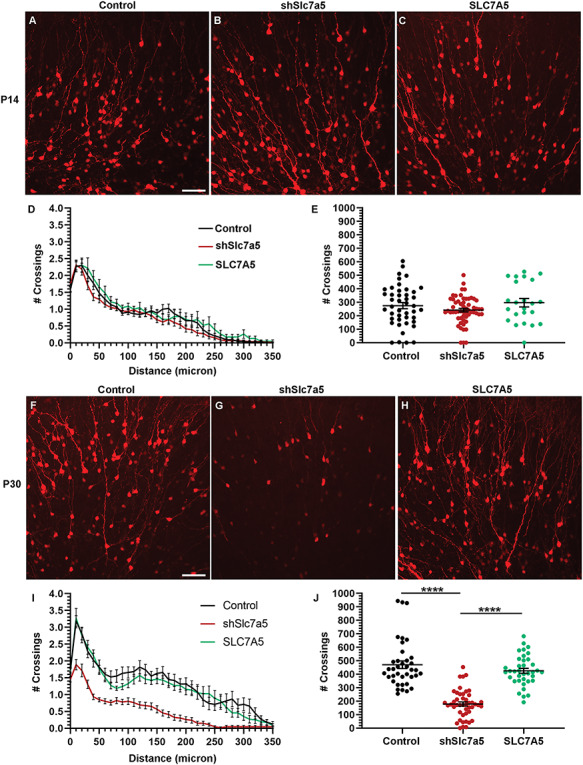
Slc7a5 is required for GC dendrite morphology. (**A**–**C**) ×20 images of P14 OB GCs from P0 electroporation of RFP and control, shSlc7a5, or SLC7A5 plasmids. (**D**) Sholl analysis of A-C. P14 control, *N* = 6, *n* = 44; shSlc7a5, *N* = 4, *n* = 58; SLC7A5, *N* = 3, *n* = 23. (**E**) Total number of crossings per GC for A-C. (**F–H**) ×20 images of P30 GCs from P0 electroporation of RFP and control, shSlc7a5 or SLC7A5 plasmids. (**I**) Sholl analysis of F–H. P30 control, *N* = 4, *n* = 38; shSlc7a5, *N* = 3, *n* = 46; SLC7A5, *N* = 4, *n* = 37. (**J**) Total number of crossings per GC for F–H. Scalebar = 50 μm. ^****^ = *P* < 0.0001.

Fourteen days after neuroblasts migrate away from the SVZ, they have become immature neurons and have formed immature basal and apical dendrites that extend into the GC layer of the OB (45, 46). Dendrite arborization was quantified in control, shSlc7a5 and SLC7A5 electroporated mice. P14 control GCs exhibited a morphology similar to what has previously been shown in rats and more recently *in vivo* in mice, having ~ 2.3 crossings within 50 μM of the cell soma, characteristic of basal dendrites and a singular immature apical projecting dendrite within the GC layer (Fig. 2A) (45, 47). GC dendrite complexity was quantified by tracing electroporated GCs, performing Sholl analysis, and calculating the total number of dendrite crossings per GC. No significant changes to dendrite complexity or total number of crossings were detected in shSlc7a5 or SLC7A5 GCs compared with controls at P14 (Fig. 2A–E).

Additional phases of GC apical and basal dendrite growth and maturation occur until GCs are 30 days old (45, 46, 48). In agreement, P30 GC dendrite arbors appeared more complex than at P14 (Fig. 2A and F). There were more proximal (basal) and distal (apical) dendrite crossings (Fig. 2D and I). Moreover, the total number of dendrite crossings per GC increased from P14 to P30 (*P* < 0.001) (Fig. 2E and J). In contrast, Slc7a5 knockdown dendrite arbors were critically reduced at P30 (Fig. 2G and I). Slc7a5 knockdown GCs had on average ~ 25% fewer crossings within 50 μM and apical processes were truncated (Fig. 2I). Moreover, Slc7a5 knockdown decreased the total number of dendrite crossings per neuron at P30 (*P* < 0.0001) (Fig. 2J). During the same period, SLC7A5 did not significantly change dendrite arborization further demonstrating the specificity of this effect (Fig. 2H–J). Reduced dendrite complexity and total number of crossings were verified with a second Slc7a5 shRNA (Fig. S3A and B). Taken together, these results demonstrate that Slc7a5 is required for GCs to acquire and maintain a mature dendrite arbor.

### Slc7a5 is required for developing GC survival

**Figure 3:**
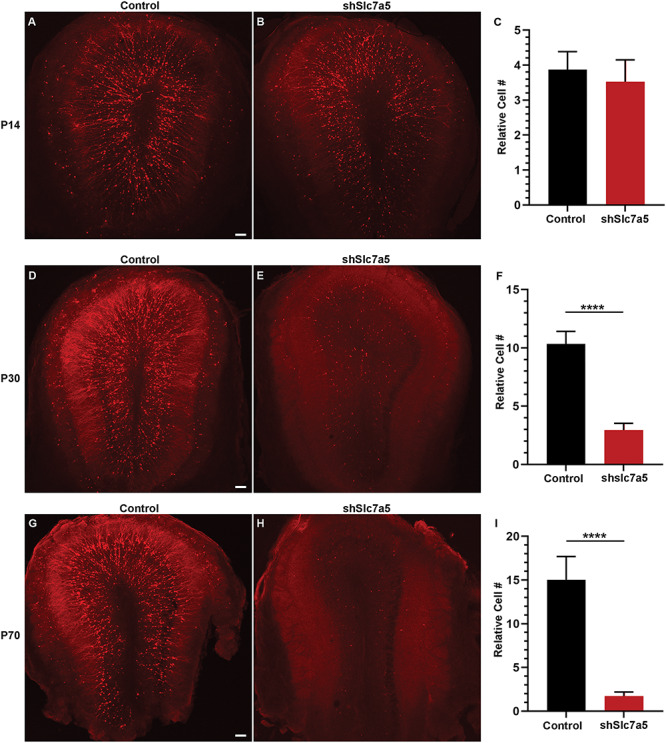
Slc7a5 is required for GC survival. (**A** and **B**) ×5 images of P14 Obs from P0 electroporation of RFP and control or shSlc7a5 plasmids. (**C**) Relative number of RFP+ GCs at P14 for control or shSlc7a5 conditions. P14 control, *N* = 6; shSlc7a5, *N* = 6. (**D** and **E**) ×5 images of P30 Obs from P0 electroporation of RFP and control or shSlc7a5 plasmids. (**F**) Relative number of RFP+ GCs at P30 for control or shSlc7a5 conditions. P30 control, *N* = 7; shSlc7a5, *N* = 8. (**G** and **H**) ×5 images of P70 Obs from P0 electroporation of RFP and control or shSlc7a5 plasmids. (**I**) Relative number of RFP+ GCs at P30 for control or shSlc7a5 conditions. P70 control, *N* = 4; shSlc7a5, *N* = 3. Scalebar = 100 μm. ^****^ = *P* < 0.0001.

During P30 morphological analysis, frequency histogram profiles highlighted that many RFP positive cells in shSlc7a5 conditions had few to no dendrite processes and on average, GCs had a smaller soma size (Fig. S3C and D). Initially, GC proximal dendrites found in the GC layer receive GABAergic input from surrounding GCs around P14 (46, 48). By P30, dendrites reach the external plexiform layer to receive glutamatergic input from tufted and mitral cells that is critical for activity-dependent survival (45, 46, 48). Therefore, the extent that Slc7a5 GCs survive with an immature (P14) or without a mature (P30) dendrite arbor was determined. There was no difference in the number of RFP positive GCs between control or shSlc7a5 conditions at P14 (Fig. 3A–C). This is not surprising due to the absence of morphological defects by P14 following Slc7a5 knockdown. However, by P30 a robust phenotype manifested, with 70% fewer GCs present after Slc7a5 knockdown compared with control (*P* < 0.0001) (Fig. 3D–F). By P70, the phenotype worsened, with almost no RFP labeled Slc7a5 knockdown GCs present in the OB (*P* < 0.0001) (Fig. 3G–I). These findings were also confirmed at P30 using a green fluorescent protein (GFP) plasmid to rule out the effect being caused by RFP (*P* < 0.01), as well as a second shRNA that targets Slc7a5 (*P* < 0.001) (Fig. S4). The results from these experiments demonstrate that Slc7a5 is required for GC survival.

### Slc7a5 regulation of dendrite maturation depends on mTOR pathway activity

**Figure 4:**
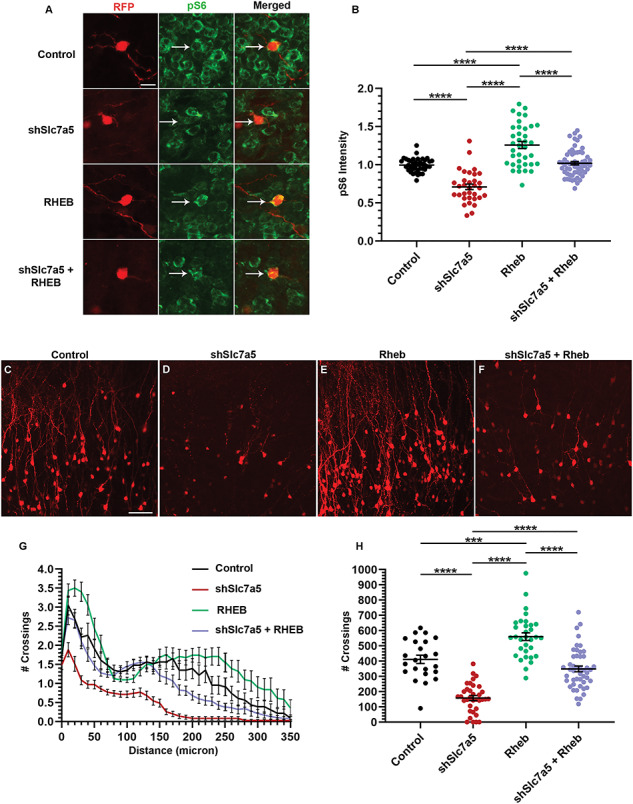
Slc7a5 regulation of dendrite morphology depends on mTOR pathway activity. (**A**) Images of P30 GCs from RFP and control, shSlc7a5, Rheb or shSlc7a5 with Rheb plasmids, stained for pS6. Scalebar = 12.5 μm. (**B**) The quantification of pS6 staining intensity for A. control, *N* = 3, *n* = 39; shSlc7a5, *N* = 5, *n* = 33; Rheb, *N* = 3, *n* = 37; shSlc7a5 and Rheb, *N* = 5, *n* = 60. (**C–F**) ×20 images of P30 GCs from P0 electroporation of RFP and control, shSlc7a5, Rheb, or shSlc7a5 and Rheb plasmids. Scalebar = 50 μm. (**G**) Sholl analysis for C-F. (**H**) Total number of crossings per GC for C-F. control, *N* = 3, *n* = 23; shSlc7a5, *N* = 4, *n* = 35; Rheb, *N* = 5, *n* = 32; shSlc7a5 and Rheb, *N* = 5, *n* = 47. ^***^ = *P* < 0.001. ^****^ = *P* < 0.0001.

Due to the fact that Slc7a5 can regulate the mTOR pathway and that the mTOR pathway regulates dendrite growth, it was tempting to speculate that the defects seen following Slc7a5 knockdown could be caused by decreased mTOR pathway activity (9, 17, 23, 25, 34). Ectopic Ras homolog enriched in brain (Rheb) binds to and activates mTORC1 in the absence of leucine (49, 50). It was hypothesized that if this occurred, over-expression of Rheb, a direct activator of mTORC1, might be able to overcome the effect of Slc7a5 knockdown. To begin to address this hypothesis, littermates were co-electroporated with RFP and control, Rheb, shSlc7a5, or shSlc7a5 and Rheb plasmids. Immunohistochemistry was subsequently performed for pS6 at P30. As expected, Slc7a5 knockdown decreased pS6 by ~ 29% (*P* < 0.0001) (Fig. 4A and B). This result confirms that Slc7a5 regulates the mTOR pathway. Also as predicted, Rheb littermates had ~ 26% more pS6 compared with control (*P* < 0.0001) (Fig. 4A and B). Notably, and as hypothesized, Slc7a5 knockdown GCs co-electroporated with Rheb had pS6 levels completely rescued compared with control (Fig. 4A and B).

Since mTOR pathway activity was rescued in Slc7a5 knockdown GCs co-electroporated with Rheb, we wondered to what extent Rheb might also mitigate dendrite defects in Slc7a5 knockdown GCs. First, dendrite morphology was measured in control or Rheb electroporated GCs. Rheb by itself increased dendrite complexity in agreement with increased pS6, similar to previous reports (*P* < 0.001) (Fig. 4C, E, G and H) (51, 52). Consistent with previous litters from Figure 1, Slc7a5 knockdown decreased GC dendrite complexity compared with control (Fig. 4C, D, G and H). In contrast, shSlc7a5 and Rheb co-electroporated dendrite complexity was partially rescued compared with Slc7a5 knockdown GCs (Fig. 4C, F and G). In addition to Sholl analysis, the total number of crossings per GC was calculated. As expected, shSlc7a5 GCs had ~ 53% fewer total crossings compared with controls, and this decrease was rescued by Rheb (*P* < 0.0001) (Fig. 4H**).** In shSlc7a5 OBs, there was a 74% reduction in GCs compared with control OBs (*P* < 0.0001) (Fig. S5). However, Rheb co-electroporation increased the amount of GCs to 220% of shSlc7a5 alone (*P* < 0.05) (Fig. S5). These results demonstrate that Slc7a5 is required for mTORC1 pathway activity, dendrite maturation, and survival.

## Discussion

Mutations in Slc7a5 and components of the mTOR pathway result in abnormal brain development and neurological manifestations. The evidence presented here supports the hypothesis that Slc7a5 regulates mTOR pathway activity and is required for neonatal OB GC dendrite maturation and survival. SVZ NSCs were electroporated neonatally with Slc7a5 shRNA or SLC7A5 to attempt to address the relative contribution of Slc7a5 to neural development. SVZ NSCs generate neuroblasts that migrate through the RMS into the OB. The majority of the neurons matures into inhibitory GCs of the GC layer and continues to mature over a 30 day period (45, 46, 48). Slc7a5 knockdown did not change the number of cells in the SVZ at P14 or P30. SVZ NSCs rapidly dilute electroporated plasmids into daughter cells, and therefore it could be argued that knockdown did not occur in the time required to see an effect. However, knockdown *in vitro* occurred within 2 days, indicating that this is not a likely possibility. Although Slc7a5 knockdown occurs rapidly, it could take a longer period of Slc7a5 depletion to exert an effect. The fact that equal numbers of NSCs retain plasmid DNA and hence were fluorescent at P14 and P30 would argue against time-dependent depletion being critical for the survival of NSCs but could affect rates of proliferation or differentiation.

Surprisingly, the quantity and morphology of Slc7a5 knockdown GCs did not significantly differ from controls at P14. At this time of development, GC dendrite arbors are immature and are able to respond electrophysiologically to GABA but not glutamate (46, 48). Robust dendrite arborization, spine formation and electrophysiological responses of GCs to glutamate occur later, at ~ 28–30 days, when dendrodendritic synapses are maturing in the external plexiform layer (45, 46, 48). The results presented here support the notion of continued GC dendrite growth into the external plexiform layer from P14 to P30.

We found that dendrites were critically malformed at P30. Mechanistically, this was associated with reduced mTOR pathway activity. Slc7a5 knockdown reduced leucine levels in Neuro-2a cells. Leucine binds to Sestrin2 to modulate mTORC1 (20). The ratio of phospho-4E-BP to total 4E-BP, a measure of mTOR, is reduced in BBB Slc7a5 knockout mice (1). In comparison, Slc7a5 null embryos had reduced pS6 potentially indicative of aberrant mTOR (9). Therefore, an alternative interpretation of the current study is that there may be a loss in the regulation of mTOR as opposed to strict loss of mTOR activation. Abnormal dendrite arborization is a shared characteristic in a range of neurodevelopmental disorders and these patients often present with neurological manifestations including epilepsy that are indicative of hyper-connectivity. As an indication of an active role of mTOR in the pathogenesis of epilepsy, mTORC1 inhibitors such as the rapalogs decrease the frequency or severity of seizures in a disease with elevated mTORC1 activity (53). In further support of the role that mTORC1 has in dendrite morphology, ectopic Rheb expression increased dendrite arborization similar to previous reports (51, 52). Moreover, co-electroporation of Rheb partially rescued the effect of Slc7a5 knockdown on survival. Ectopic Rheb binds to and activates mTORC1 in the absence of leucine (49, 50). It is therefore not surprising that Rheb rescued the effect of Slc7a5 knockdown on dendrites.

Engineered deletion of mTOR exons 1–5 causes embryonic day 6.5–7.5 lethality (54). A mutagenic screen identified an intronic mutation leading to reduced mTOR-prevented development of the telencephalon and led to death by embryonic day 11 (55). The phenotype of mutant mTOR mirrors the phenotype of a group of Slc7a5 knockout mice (9). This is similar to conditional mTOR deletion using nestin promoter-driven causes recombination (CRE), which causes microcephaly and a thinning of the cortical plate by embryonic day 15 (56). Microcephaly in the mTOR conditional mice is correlated with reduced progenitor proliferation and neurogenesis. However, mTOR is present within two complexes, mTORC1 and mTORC2. Therefore, to what extent mTORC1, mTORC2 or both are responsible for mTOR deletion phenotypes is not clear. Surprisingly, nestin-CRE deletion of Rictor or Raptor, which are the components of mTORC2 and mTORC1 respectively, phenocopy nestin-CRE deletion of mTOR (57, 58). Conditional Raptor deletion also reduces progenitor proliferation, but also reduces dendrite growth of neurons and induces robust apoptosis within cortical plate neurons (58). However, these mice also have robust changes in gliogenesis. Nestin-CRE Rictor deletion similarly produces neurons with reduced dendrite arbors (57). Thus, both mTORC1 and mTORC2 appear to be required for proper dendrite morphology. Perhaps, more pertinent to this paper is the observation that mTORC1 activity is high within the SVZ-RMS-OB axis, including in OB GCs where translation becomes highly selective (59). mTORC1 knockdown in NSCs prevents neuronal differentiation and provides evidence of the importance of mTORC1 regulation in neural differentiation (60). Indeed, rictor, raptor and mTOR knockdown as well as mTOR or raptor deletion by neonatal electroporation of SVZ NSCs reduces OB GC mTORC1 activity and dendrite growth (35). These results are consistent with previous observations that removal of negative regulators of mTOR, such as TSC1/2, increase dendrite growth (61). Taken together, the loss of Slc7a5 resulting in reduced or abnormal mTOR activity is consistent with current models of how mTORC1 regulates OB GC maturation.

A limitation of this study is that although Slc7a5 transports leucine, numerous other amino acids, dopamine and thyroid hormones are also transported (14–16, 62, 63). Although the current study demonstrates that Slc7a5 is required for leucine transport, it does not address whether other molecules are involved in the requirement for Slc7a5 or whether compensatory changes may contribute to our findings and further experimentation is required in future studies. A major question that arises from these studies is why certain cells do not require Slc7a5, although the existence of other transporters and mechanisms of acquiring nutrients such as autophagy or micropinocytosis are possible. In summary, the results of this study highlight the existence of a transient and cell-specific requirement for Slc7a5 to regulate the mTOR pathway and GC dendrite growth.

## Materials and Methods

### Animals

Clemson University Institutional Animal Care and Use Committee approved all experiments performed, and all guidelines set forth by the Clemson University Institutional Animal Care and Use Committee and NIH Guide for the Care and Use of Laboratory Animals were followed. Charles River Laboratories provided timed pregnant CD-1 mice. Mice were housed under pathogen-free conditions with a 12-h light/dark cycle and fed *ad libitum*.

### Plasmids

CAG-Rheb (a kind gift from Dr Seonhee Kim) and CAG-tdTomato (Addgene # 83029, a kind give from Dr Angelique Bordey) have been described elsewhere (64, 65). CAG-SLC7A5 was synthesized by a commercial vendor (SignaGen Laboratories). shSlc7a5 (Sigma Aldrich, TRCN0000443348 and supplemental TRCN0000426198), control (Sigma Aldrich empty vector), CAG-GFP (Addgene #11150) and CMV-myc-Slc7a5 (Slc7a5 NM_011404 Mouse Tagged ORF Clone, OriGene) plasmids are available through the indicated commercial vendors.

### Neuro-2A cell culture and transfection

Neuro-2a mouse neuroblastoma cells (American Type Culture Collection CCL-131) were maintained in Dulbecco’s modified Eagle’s medium (DMEM), 10% fetal bovine serum, and penicillin/streptomycin in tissue culture-treated polystyrene flasks (Falcon; BD Biosciences Discovery Labware) in a 37°C incubator with 5% CO_2_. Neuro-2a cells were passaged at a density of 4 ° 10^5^ cells into six well plates 24 h before transfection. PolyJet (SignaGen Laboratories) was mixed at a 3:1 ratio with plasmid DNA in DMEM and ~1 μg of plasmid was added to each well.

### Neonatal electroporation

Timed pregnant CD-1 mice were delivered from Charles River Laboratories, acclimated to the animal facility and the date of birth was recorded. Neonatal mice (P0-P1) were injected with equal amounts, concentrations and volumes of DNA plasmids (~1.3–2.0 μg/μl) diluted in phosphate buffered saline (PBS) with 0.1% fast green. DNA was injected into the lateral ventricles illuminated with a fiber optic light source, and delivered using a ultraviolet sterilized borosilicate glass micropipette generated from capillary tubes as previously described (66). Borosilicate capillary tubes were pulled with a P97 Sutter micropipette puller with the settings. Tweezer electrodes (model 520; BTX) were rinsed in 0.9% saline solution and placed on the heads of the pups, and five, 100-volt square pulses of 50 ms duration with 950-ms intervals were applied using a pulse generator (ECM830; BTX).

### Image analysis

Images (×20) were uploaded and analyzed using FIJI (ImageJ 1.5 g). Simple neurite tracer plug-in was used to trace dendrite processes of 23–58 RFP positive cells from 3–6 mice per condition. Sholl analysis was performed at 10 μm intervals to quantify both apical and basal dendritic arborization using the Sholl plug-in. A total number of dendritic crossings were calculated by taking the sum of crossings at 1 μm intervals for each traced neuron and averaging the total number of crossings per neuron in each condition. The number of labeled OB GCs from 3–8 mice was quantified by using FIJI cell counter plugin.

Images (×20) of RFP positive OB GCs stained by immunohistochemistry were uploaded to FIJI (ImageJ 1.5 g). The freehand selection tool was used to trace a region of interest (ROI) on electroporated cells and record a mean gray value to quantify the staining intensity of pS6 in indicated conditions. Pairwise comparison of staining in non-electroporated cells in the same Z section was performed. Staining was performed on 3–5 mice per condition and 33–60 cells per condition.

### Slice preparation and immunohistochemistry

Pentobarbital (50 mg/kg) was administered by intraperitoneal injection to anesthetize mice before decapitation. The brain was dissected in room temperature PBS, transferred to 4% paraformaldehyde (in PBS), and incubated overnight at 4°C. Next, brains were washed in PBS and mounted in low melt agarose (3%). A Leica VTS 1000 vibratome was used to slice the brains coronally in 300 μm sections. Immunostaining was performed with slices in 24 well plates. Free-floating sections were blocked in PBS containing 0.1% Triton X-100, 0.1% Tween-20 and 2% BSA and incubated in primary antibody pS6 (rabbit anti-pS6; 1:1000; cell signaling technology; Ser 240/244, 61H9, #4838), or SLC7A5 (rabbit anti-SLC7A5; 1:1000; Cell Signaling Technology; #5347) overnight at 4°C. After three washes in PBS containing 0.1% Tween-20, slices were incubated with the appropriate secondary antibody (Alexa Fluor series at 1:500 [Invitrogen]) overnight at 4°C. TO-PRO-3 (invitrogen) was used to counter-stain sections. Each staining was replicated on 4–6 mice per condition. Images were acquired on a spectral confocal microscope (Leica SPE) with a ×20 dry objective (N.A. 0.75). Low-magnification images were acquired with a ×5 dry (N.A. 0.15) objective.

### Western blotting

Samples were collected in 2% SDS in RIPA buffer containing protease and phosphatase inhibitor cocktail. Lysates were briefly sonicated on ice and centrifuged at 13 200 rpm for 10 min in a tabletop Eppendorf 5414 centrifuge. Proteins were resolved by standard electrophoresis conditions on 10–12% polyacrylamide precast mini-Protean TGX gels and transferred to polyvinylidene difluoride (PVDF) membranes. Membranes were rinsed in Tris-buffered saline (TBS-T, 0.1% Tween 20) for 5 min at room temperature and subsequently blocked in 5% weight/volume nonfat milk in TBS-T for 1 h at room temperature. Samples were incubated for 1 h at room temperature or overnight at 4°C with the following antibodies: rabbit anti-SLC7A5 (1:1 000; Cell Signaling Technology; #5347), rabbit anti-Myc-tag (1:1 000; Cell Signaling Technology; 71D10, #2278), and rabbit anti-Akt (pan) (1:1 000; Cell Signaling Technology; 11E7, #4685). Following an additional five rinses each of 10 min in TBS-T, samples were incubated for 1 h at room temperature with donkey or goat anti-rabbit antibodies in blocking buffer and then subjected to four 15-min washes in TBS-T and visualized using Bio-Rad Chemidoc MP imaging system. PVDF membranes were stripped for 5–15 min at room temperature using Restore Western Blot Stripping Buffer (#21059, Thermo Fisher Scientific) before probing for loading controls.

### Leucine Assay

Neuro-2A cells (4 × 10^5^) were seeded into six well plates and transfected 24 h later with DNA plasmids. After 48 h, cells were lysed and leucine levels were measured by performing a leucine assay following manufacturer instructions (Sigma-Aldrich, MAK003). Lysate (10 μl) was added to a 96 well plate and diluted to 50 μl using assay buffer. Next, 46 μl of assay buffer was added to each well, along with 2 μl of branched-chain amino acid enzyme mix and substrate mix for a final volume of 100 μl per well. Blanks were performed by mixing lysate with assay buffer and substrate mix but omitting enzyme. The blank absorbances were subtracted from lysate absorbances. A standard curve was generated with pure leucine Readings were acquired using a Synergy H1 Hybrid Reader. Each sample was measured in duplicate.

### bDNA FISH

bDNA FISH was performed using the Quantigene ViewRNA ISH Cell assay according to the manufacturer’s instructions. Briefly, 4% paraformaldehyde-fixed tissue that was washed three times in PBS and permeabilized with Detergent Solution QC followed by two rinses with PBS. Tissue was incubated in Protease QS solution at 40°C for 60 min. Tissue sections were then incubated with Probe Set Diluent QF with or without Slc7a5 probe set type 4 (Alexa Fluor 488) for 3 h at 40°C. Tissue was washed in 2 ml wash buffer for 5 min two times and then incubated for 30 min at 40°C with PreAmplifier Mix and Amplifier Diluent QF pre-warmed to 40°C. Samples were washed three times with 2 ml wash buffer for 5 min each wash. Next, tissue was incubated with 400 ml of the Label Probe Mix diluted in Label Probe Diluent QF (pre-warmed to 40°C) for 30 min at 40°C. Samples were then washed three times in 2 ml wash buffer for 10 min, counterstained and mounted in ProLong Gold Antifade mounting reagent.

### RT-PCR

OBs from mice were removed from ~P30 mice and subjected to TRIzol RNA isolation according to the manufacturer’s protocol. Briefly, An OB was added to ~ 250 μl of TRIzol reagent and passed through a 22-gage needle into 1.5 ml reaction tubes. Samples were incubated at 4°C for 10 min. 50 μl of chloroform was added to each tube, vortexed and incubated for 10 min at 4°C. Samples were centrifuged for 10 min at 12 000 x g at 4°C and the upper aqueous phase was transferred to a fresh tube. In total, 125 μl ice-cold isopropanol was added to the aqueous phase, samples incubated at 4°C for 10 min and then centrifuged for 10 min at 12 000 x g at 4°C. Supernatant was discarded and RNA pellets washed in 250 μl of 75% ethanol, vortexed and centrifuged for 5 min at 7500 x g. Samples were washed a total of three times and the RNA pellet was air dried for 10 min and resuspended in 50 μl of RNAase-free water before determining concentration and purity. For RT-PCR, RNA was combined with deoxynucleotide triphosphates, random primers (invitrogen) and RAase/DNase-free H_2_O. Samples were heated for 5 min at 65°C and then rapidly chilled. Dithiothreitol, RAase out and SuperScript III were then added to each sample and reverse transcribed. cDNA was subjected to PCR using primers to Slc7a5 and Platinum Taq polymerase.

### Statistics

Data were graphed and analyzed with GraphPad Prism software (Version 8.2.0, GraphPad Software Inc.). Statistical significance was determined by Student’s *t*-test (cell number analysis), one-way analysis of variance (ANOVA) (ROI and total number of crossings analysis) with multiple comparisons test, or two-way ANOVA with Tukey’s multiple comparisons test (Sholl analysis). All experiments were performed on 3–8 mice per condition per time point. *N* (number of cultures or mice) and *n* (number of cells) are listed where applicable.

## Supplementary Material

Supplementary_Figure_1_ddaa186

Supplementary_Figure_2_ddaa186

Supplementary_Figure_3_ddaa186

Supplementary_Figure_4_ddaa186

Supplementary_Figure_5_ddaa186

Supplementary_Figure_Legends_ddaa186

## Abbreviations

SLC7A5, Solute carrier family 7 member 5; mTOR, mammalian Target of Rapamycin; mTORC1, mammalian target of rapamycin complex 1; mTORC2, mammalian target of rapamycin complex 2; TSC1, tuberous sclerosis complex 1; TSC2, tuberous sclerosis complex 2; Rheb, Ras homolog enriched in brain; eIF4E, eukaryotic initiation factor 4E; S6, ribosomal S6 subunit; shRNA, short hairpin RNA; ISH, *in situ* hybridization; FISH, fluorescent *in situ* hybridization; RT-PCR, reverse transcription polymerase chain reaction; SVZ, subventricular zone; NSC, neural stem cell; RMS, rostral migratory stream; OB, olfactory bulb; GC, granule cell; BBB, blood–brain barrier; TBS, tris-buffered saline; PBS, phosphate buffered saline.
