# Supplementary figures and images for: The amino acid transporter Slc7a5 regulates the mTOR pathway and is required for granule cell development
Source: Hum Mol Genet. 2020 Aug 21;29(18):3003–13. doi: 10.1093/hmg/ddaa186 (PMC7645712; doi:10.1093/hmg/ddaa186)

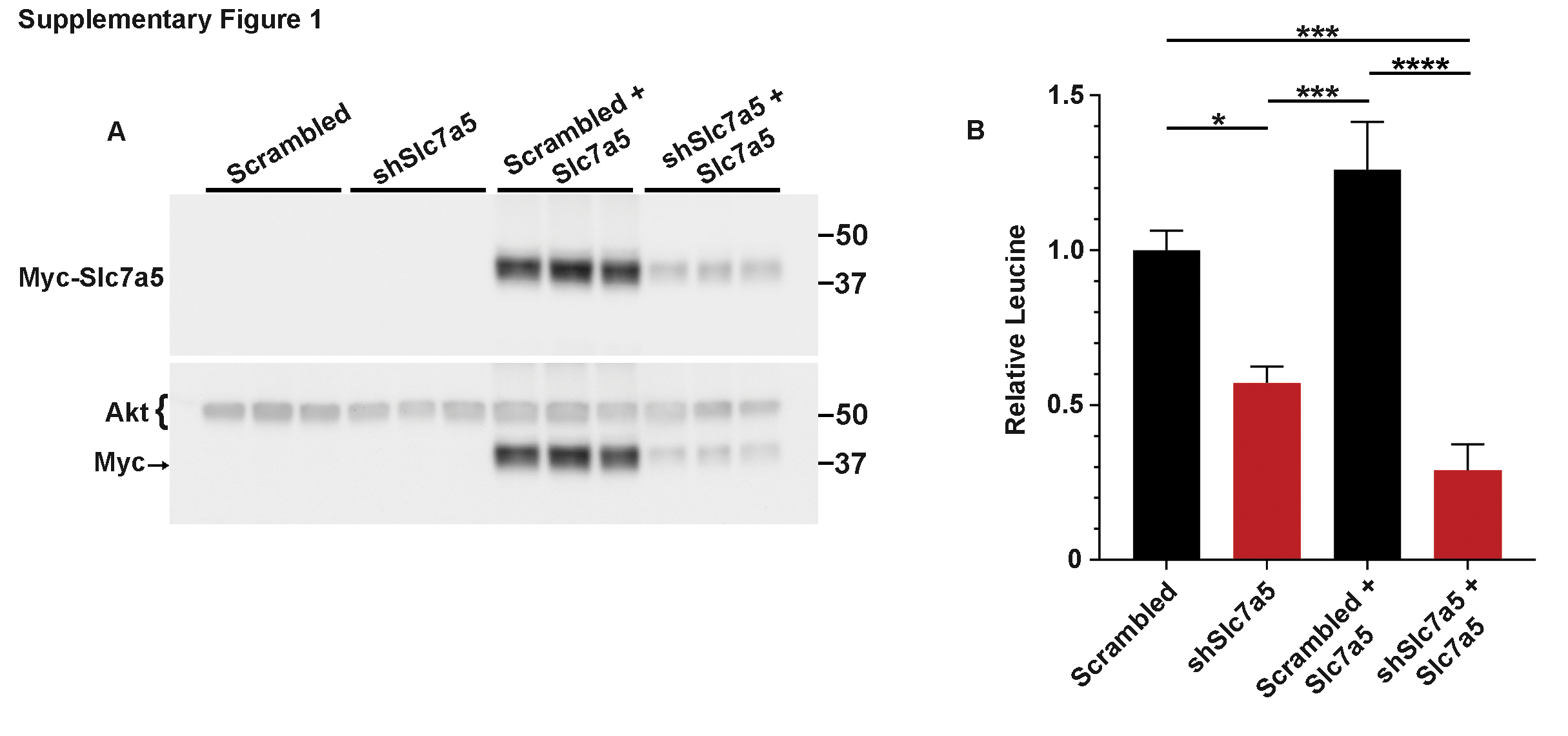

Supplement: Supplementary_Figure_1_ddaa186 [file supplementary_figure_1_ddaa186.png]

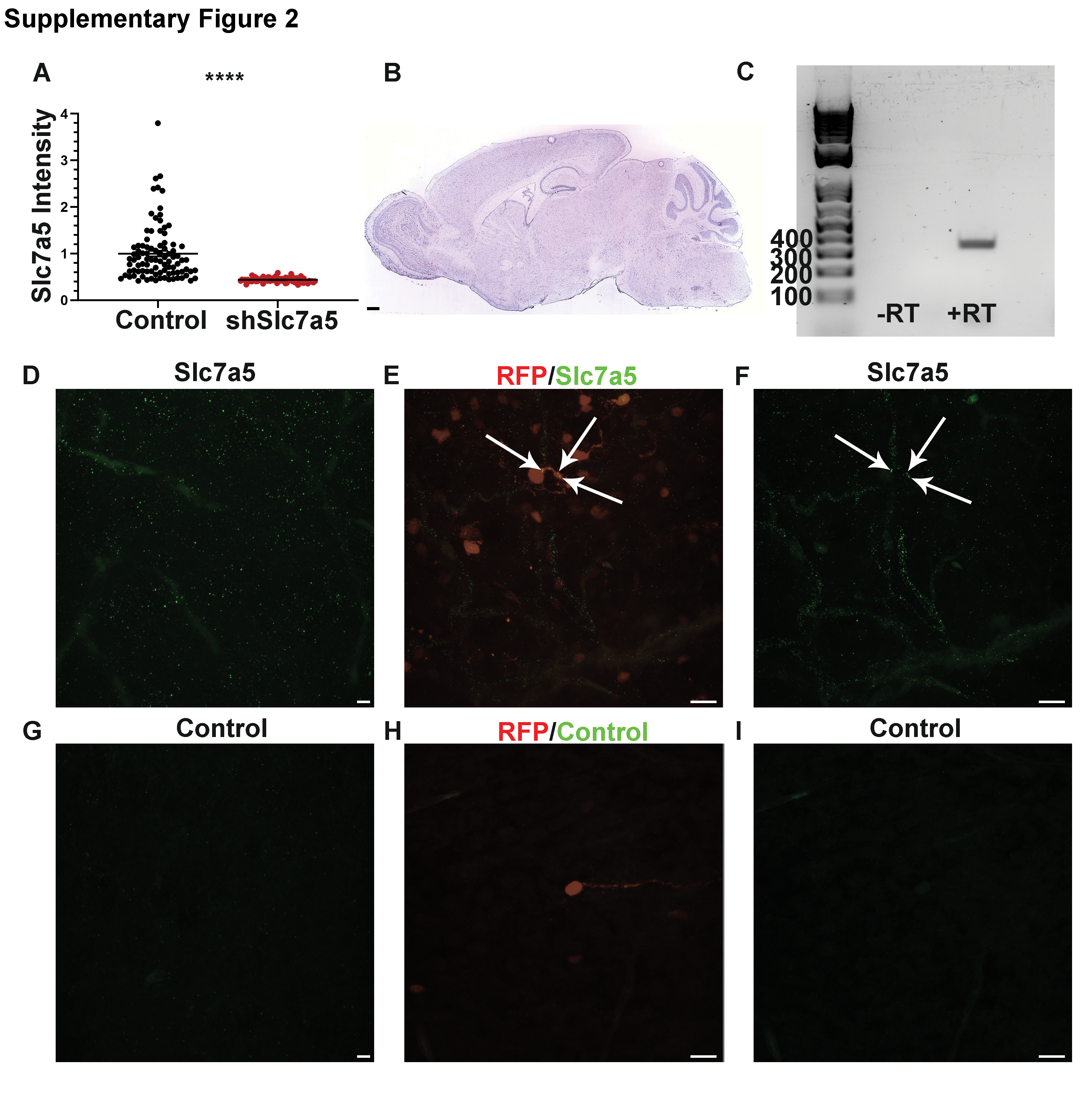

Supplement: Supplementary_Figure_2_ddaa186 [file supplementary_figure_2_ddaa186.png]

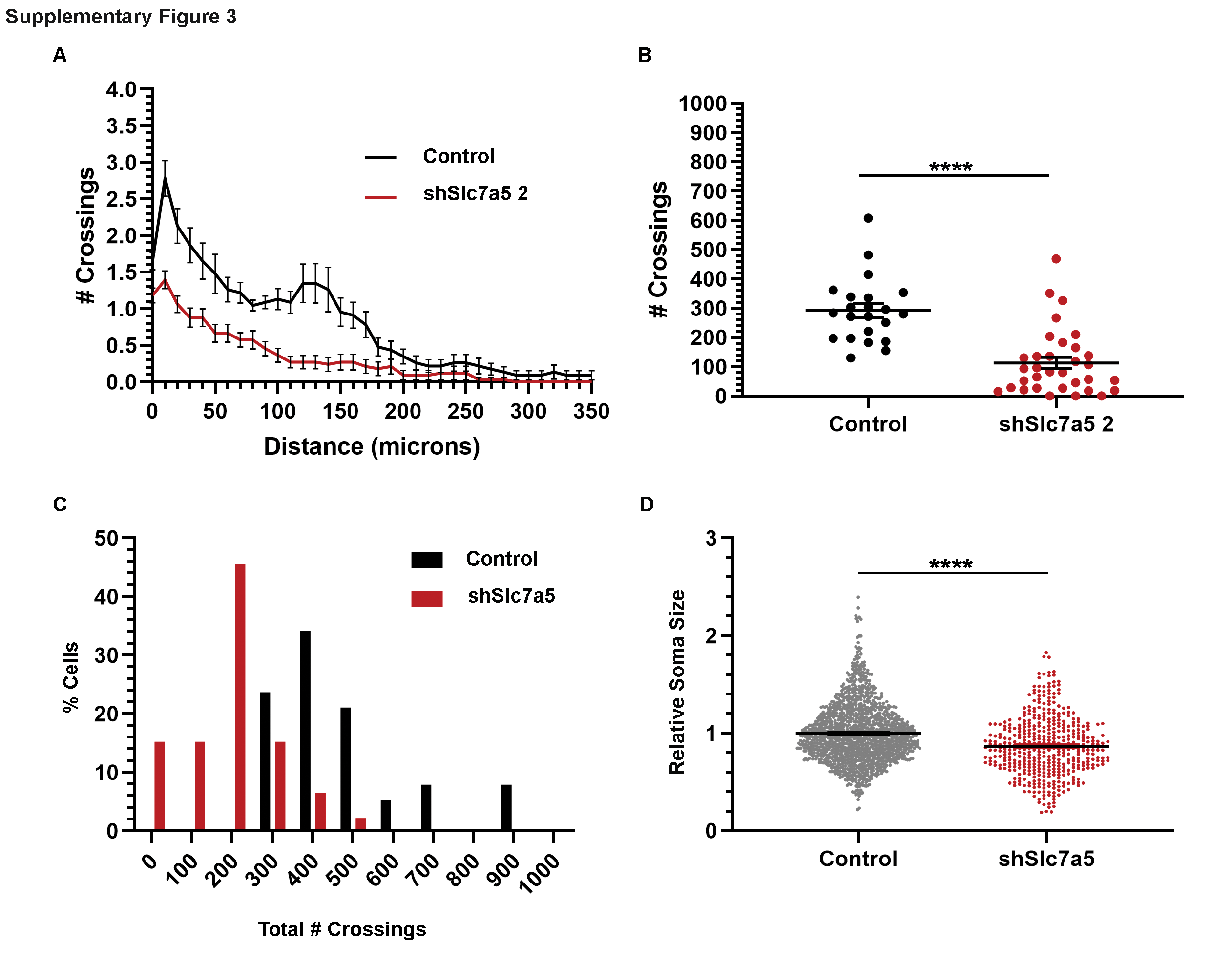

Supplement: Supplementary_Figure_3_ddaa186 [file supplementary_figure_3_ddaa186.png]

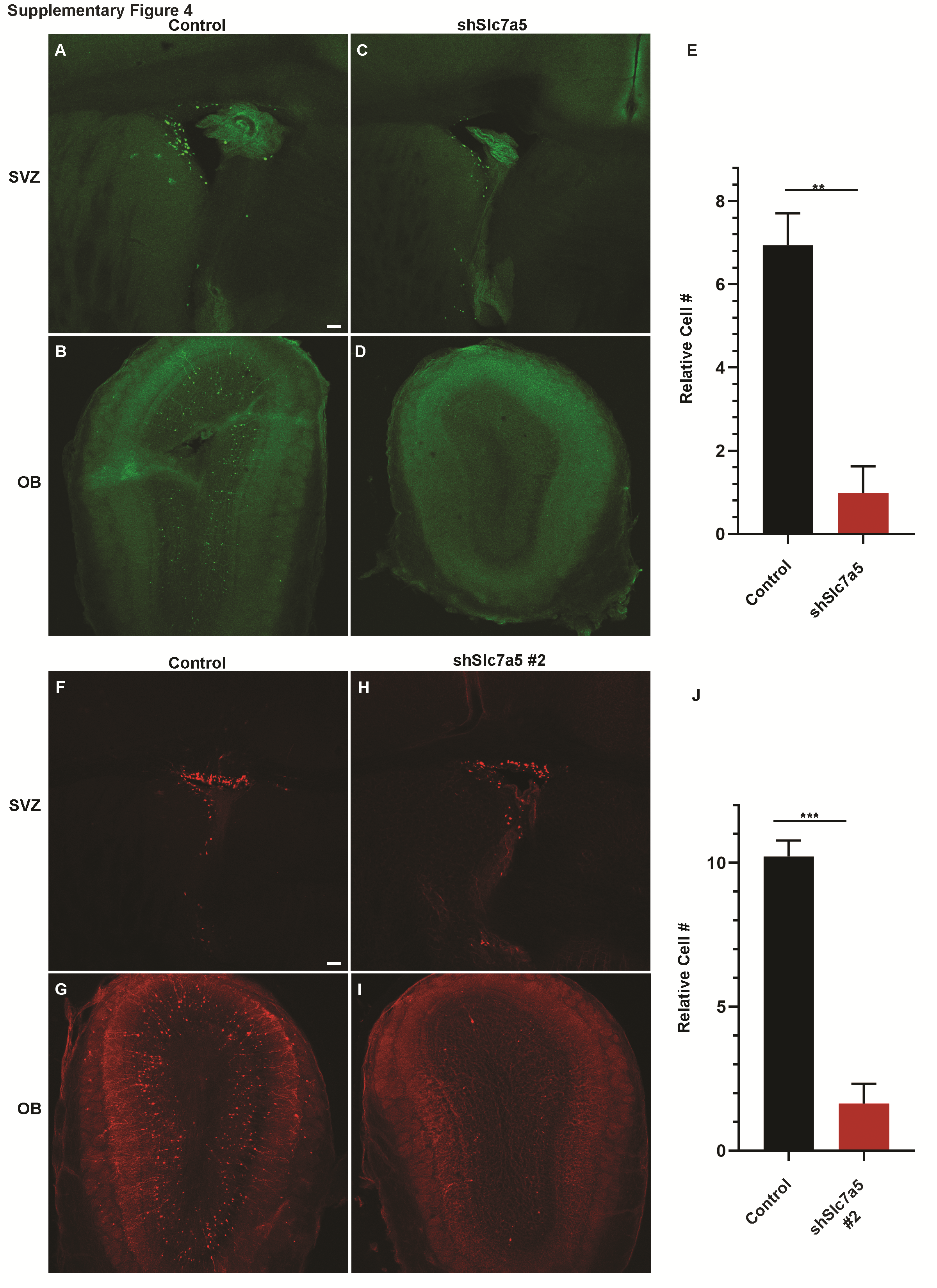

Supplement: Supplementary_Figure_4_ddaa186 [file supplementary_figure_4_ddaa186.png]

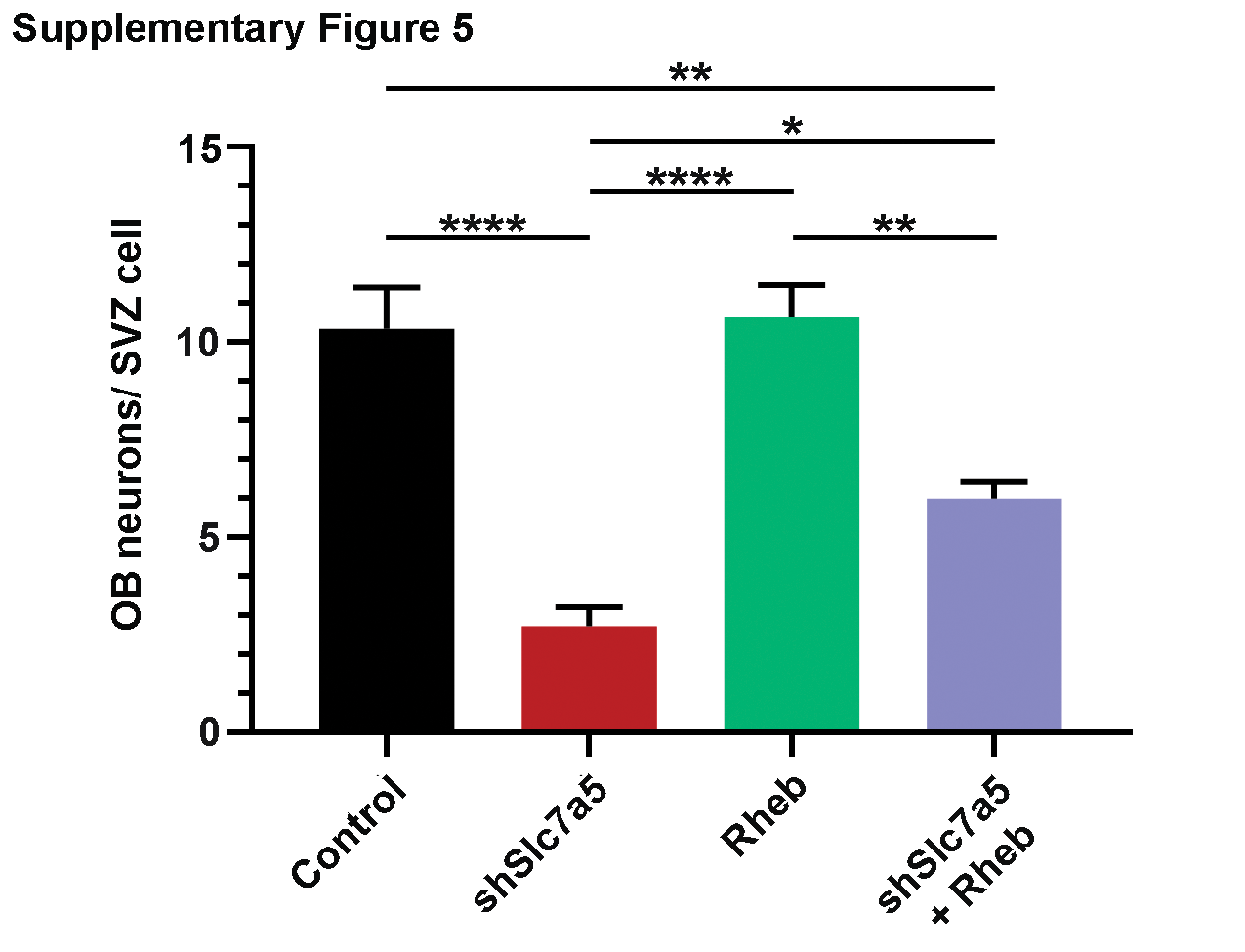

Supplement: Supplementary_Figure_5_ddaa186 [file supplementary_figure_5_ddaa186.png]
