# Supplementary material for: The amino acid transporter Slc7a5 regulates the mTOR pathway and is required for granule cell development
Source: Hum Mol Genet. 2020 Aug 21;29(18):3003–13. doi: 10.1093/hmg/ddaa186 (PMC7645712; doi:10.1093/hmg/ddaa186)
Supplement: Supplementary_Figure_Legends_ddaa186 [file supplementary_figure_legends_ddaa186.pdf]

Supplementary Figure Legends

Supplementary Figure 1. *Confirmation of Slc7a5 shRNA specificity and leucine transport.*
A. Western blot for myc from lysates of Neuro-2a cells transfected with control shRNA, shSlc7a5, control shRNA with myc-Slc7a5, or shSlc7a5 and myc-Slc7a5 plasmids (N=3, 3, 3, 3). Westerns were stripped and re-probed for total Akt as a loading control. **B.** Leucine concentrations were measured in lysates from A. and demonstrate a reduction in leucine concentration following Slc7a5 knockown. N=3, 3, 3, 3 ****=P<0.0001

Supplementary Figure 2. *Slc7a5 knockdown and OB GC expression.*
A. Quantification of Slc7a5 staining at P30 in control (SLC7A5, empty vector, and RFP) (n=92) or shSlc7a5 (SLC7A5, shSlc7a5, and RFP) electroporated cells (N=3, n=93). **B.** *In situ* hybridization of Slc7a5 in a P56 sagittal brain section from the Allen Mouse Brain Atlas. Image credit: Allen Institute. **C.** RT-PCR of mRNA from P30 OB. **D.** bDNA FISH for Slc7a5 mRNA (green spots) OB. (N=3) **E.** Overlap of RFP positive OB GCs (red) from P0 electroporations subjected to bDNA FISH (**F**) for Slc7a5 mRNA (green) (N=3). Arrows point to Slc7a5 mRNA puncta staining in electroporated GC. **G.** Control label probe only for bDNA FISH in a section from the same brain shown in D. **H.** Overlap of RFP positive OB GCs (red) from P0 electroporations subjected to bDNA FISH (**I**) for control label probe only in a section from the same brain as E-F. Arrows indicate overlap of Slc7a5 in a GC. ****=P<0.0001

Supplementary Figure 3. *Slc7a5 knockdown GC dendrites, frequency histogram profile, and cell size.* **A.** Sholl analysis of P30 neurons following electroporation at P0 with RFP and a second shRNA (shSlc7a5 2) to Slc7a5 or control plasmids (N=3, 3). **B**. Total number of crossings per GC for A. (shSlc7a5 2, N=3, n=33 vs control, N=3, n=22). **C.** Frequency histogram profile of total dendrite crossings per GC at P30 following electroporation at P0 of control (N=4, n=38) or shSlc7a5 (N=3, n=46) plasmids. **D.** Cell size analysis of GCs at P30 following electroporation at P0 of control (N=4, n=1326) or shSlc7a5 plasmids (N=3, shSlc7a5, n=450). ****=P<0.0001.

Supplementary Figure 4. *Validation of GC death with an additional* Slc7a5 *shRNA.*
A-D. 5x P30 Obs or SVZs from mice electroporated with GFP and control or shSlc7a5 plasmids. control, N=4; shSlc7a5, N=3. **E**. Quantification of A-D. **F-I**. 5x P30 Obs or SVZs from mice electroporated with RFP and control or a second alternative shRNA to Slc7a5. Control, N=3; shSlc7a5, N=3. **J**. Quantification of F-I. SVZ=Subventricular zone. **=P<0.01. ***=P<0.001.

Supplementary Figure 5. Relative number of RFP+ GCs at P30 for control (N=7), shSlc7a5 (N=8), Rheb (N=4), and shSlc7a5 and Rheb (N=5). *=P<0.05. **=P<0.01. ****=P<0.0001
